# Biodegradable Poly(Lactic Acid) Nanocomposites for Fused Deposition Modeling 3D Printing

**DOI:** 10.3390/nano10122567

**Published:** 2020-12-21

**Authors:** Madison Bardot, Michael D. Schulz

**Affiliations:** Department of Chemistry and Macromolecules Innovation Institute, Virginia Tech, Blacksburg, VA 24061, USA; mbardot2@vt.edu

**Keywords:** 3D printing, poly(lactic acid) (PLA), additive manufacturing (AM), fused deposition modeling (FDM), cellulose, carbon nanoparticles

## Abstract

3D printing by fused deposition modelling (FDM) enables rapid prototyping and fabrication of parts with complex geometries. Unfortunately, most materials suitable for FDM 3D printing are non-degradable, petroleum-based polymers. The current ecological crisis caused by plastic waste has produced great interest in biodegradable materials for many applications, including 3D printing. Poly(lactic acid) (PLA), in particular, has been extensively investigated for FDM applications. However, most biodegradable polymers, including PLA, have insufficient mechanical properties for many applications. One approach to overcoming this challenge is to introduce additives that enhance the mechanical properties of PLA while maintaining FDM 3D printability. This review focuses on PLA-based nanocomposites with cellulose, metal-based nanoparticles, continuous fibers, carbon-based nanoparticles, or other additives. These additives impact both the physical properties and printability of the resulting nanocomposites. We also detail the optimal conditions for using these materials in FDM 3D printing. These approaches demonstrate the promise of developing nanocomposites that are both biodegradable and mechanically robust.

## 1. Introduction

Additive manufacturing (3D printing) enables rapid prototyping, convenient customization, and unique capabilities, while democratizing the manufacturing process in ways that are only just beginning to be leveraged on a large scale [1,2,3]. These burgeoning manufacturing trends, however, also intersect with growing concerns about the ecological impact of the materials used in manufacturing. As pollution from plastic waste grows worldwide, developing materials that are biodegradable and bio-renewable becomes increasingly important [4,5,6]. Unfortunately, most materials commonly used for 3D printing are neither.

One of the most common methods of 3D printing—fused deposition modeling (FDM), also known as fused filament fabrication—uses layer-by-layer addition of polymeric materials to form a completed piece. This addition is facilitated by computer-aided design, which instructs the printer where to add polymer [7]. FDM involves drawing a filament through a heated extrusion head, which deposits the molten polymer onto a bed where the 3D-printed part forms [7,8]. The FDM process requires specific parameters for drawability and processability that influence not only the filament production but also the layer deposition during printing [9]. For viable printing, the extruded material must have a low melting temperature and fast solidification time [7].

The printability and strength of printed parts also relies on good adhesion between layers and a homogeneous distribution of any additives [10]. Uniform distribution of additives ensures that agglomerates do not clog the printing apparatus or cause weak points in the printed material [11]. Compounding the complex nature of FDM 3D printing, various printing parameters—raster angle, raster width, layer thickness, build orientation, infill density and pattern, feed rate, and air gap—also have large effects on the quality and mechanical properties of FDM-printed parts [12,13,14,15,16,17].

Several polymers can be readily printed by FDM including acrylonitrile butadiene styrene (ABS), polycarbonate (PC), and poly(lactic) acid (PLA) [18]. Of these polymers, only PLA is biodegradable, and of the limited number of biodegradable polymers evaluated for 3D printing, PLA is by far the most common. However, its limited mechanical properties hinder its use in many applications, particularly in industrial settings. Additionally, PLA is insulating, which precludes its use in conducting parts [19]. To address each of these issues, various additives have been incorporated into PLA to increase its strength and conductivity. In this review we delve into different additives that enhance mechanical, thermal, or electrical properties while maintaining the biodegradability of the resulting PLA/additive nanocomposite. These nanocomposites exemplify the possibility of developing biodegradable materials while maintaining key physical properties required for industrial applications.

## 2. Poly(Lactic Acid) (PLA): 3D Printing Properties

**Figure 1:**
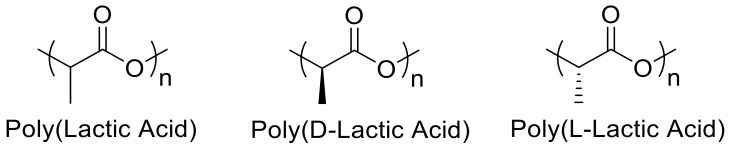
Poly(lactic acid) conformers.

PLA is popular for 3D printing due to its affordability, renewability (e.g., derived from corn or sugar cane), and biocompatibility [7,20,21]. PLA contains repeating lactic acid units as depicted in Figure 1, and can include both D- and L-stereoisomers, or be enantiomerically pure (e.g., PLLA contains only L stereocenters). PLA in conjunction with FDM produces 3D scaffolds that degrade via hydrolysis to lactic acid, a common biologically produced molecule [22,23]. PLA biodegradation depends on pH (degrading faster in highly acidic or basic media), temperature, autocatalytic behavior (catalysis by the lactic acid formed during degradation), and the degree to which water enters the matrix [23]. PLA also retains good mechanical strength while remaining processable through melt mixing, solution mixing, injection molding, and 3D printing [21]. However, several drawbacks limit its industrial use such as brittleness, poor thermal stability, low crystallinity, low elongation at break, poor impact strength, low heat-distortion temperature, and limited drawability [9,21,24]. These drawbacks, especially the slow crystallization, deter the replacement of fossil-based thermoplastics with PLA [25]. To increase the functionality of PLA, researchers have introduced additives such as cellulose, metals, carbon, continuous fibers, and others to modulate properties such as thermal conductivity, electrical conductivity, mechanical strength, viscosity, and degradation time [9,26].

**Figure 2:**
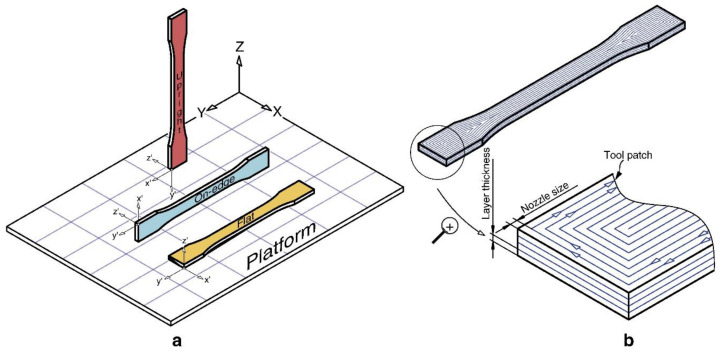
(**a**) Flat, on-edge, and up right printing orientation of tensile bars. (**b**) Processing parameters of tensile bars. Reproduced with permission from [27], Elseveir, 2017.

FDM 3D printing parameters affect the printed materials and printing process. Manufacturing time and costs are directly linked to such parameters: production time (and therefore cost) decreases with increasing feed rate and layer thickness. Chacón et al. investigated the effects of these parameters on the properties of neat PLA FDM-printed tensile bars as depicted in Figure 2 [27]. Increasing layer thickness increased tensile and flexural strength for upright printed tensile bars. However, on-edge and flat printed tensile bars had only slight differences in the tensile and flexural strength [27,28]. Decreasing feed rate decreased tensile and flexural strength in upright samples but had limited effects on materials produced by on-edge and flat printing orientations. For all samples, the ductility decreased as layer thickness increased. Overall, on-edge orientation produced the best mechanical performance, ductility, and stiffness. Moreover, if an on-edge orientation is used, high layer thickness and low feed rate maximize ductility [27]. Tensile properties increased with infill density regardless of infill pattern, including concentric infill patterns (which produced the greatest tensile strength), grid patterns, and tri-hexagonal patterns [29,30].

Thermal processing conditions also play a key role in sample material properties. For example, samples made with low build platform temperatures have increased mechanical properties, increased interfacial strength, larger crystal size, and lower crystallinity [31,32,33]. Additionally, post-printing annealing at 120 °C removes internal stresses, changes the crystallinity, removes the δ form (a more disorganized crystalline structure), and improves the storage modulus, especially in samples with large layer thickness [31]. The surface structure and friction can also be modulated by changing printing parameters [34].

Traditionally, PLA is sold as filaments for FDM with colorants and additives already incorporated. These additives can have profound effects on the properties of the resulting printed material. For example, Cicala et al. observed a marked difference in elasticity among various commercial PLA samples, demonstrating the effect of different additives on mechanical properties [35]. Importantly, Cicala et al. determined that polymers with high viscosity print with increased precision because of their resistance to flow after printing, which allows them to hold their shape and minimize voids between printed layers [35]. Cuiffo et al. investigated commercial PLA samples with calcium carbonate additives and found that the CaCO_3_ concentrated in the voids of the 3D-printed materials after FDM printing [36]. Additionally, these PLA samples underwent minor chemical reactions during the FDM process, as shown by changes in the Fourier transform infrared (FTIR) stretches for the C=O, C–O–C, -CH_3_, and -OH functional groups, and by deviations in the cold crystallization (T_cc_) and melting (T_m_) temperatures [36].

Neat PLA has a T_g_ of 55–65 °C [36,37] and T_m_ of 173–178 °C [36], which enables FDM printing. While the thermal and mechanical properties of PLA are appropriate for FDM 3D printing, they are often inappropriate for many applications, which require a different set of properties. To achieve such materials, while maintaining the renewability and degradability of PLA, new additives have been explored beyond those typical of commercial samples. These additives have resulted in a range of new materials that show promise for applications beyond the capabilities of neat PLA.

## 3. Cellulose-Based Additives

**Figure 3:**
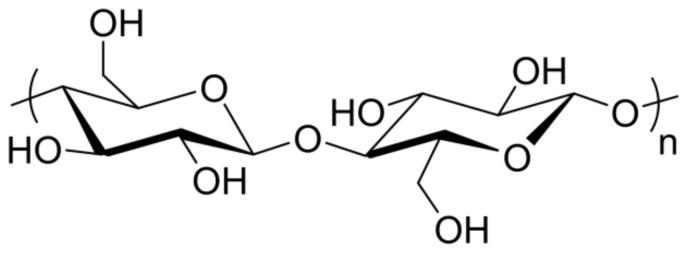
Cellulose chemical structure.

An increasingly attractive nanofiller for PLA-based materials is cellulose. Cellulose, a biodegradable material, is the most abundant natural polymer shown in Figure 3, and forms a fully biodegradable nanocomposite when incorporated into PLA [7]. Cellulose nanocrystals (CNCs), in particular, attract attention as an additive because of their strength, low weight, transparency, and biocompatibility [24]. While the addition of cellulose may increase mechanical properties, the crystallinity and hydrophobicity of CNCs hinder their incorporation into the PLA matrix. The crystallinity of CNCs, approximately 54–88%, accounts for their high tensile modulus (105–250 GPa), which is greater than that of both glass fiber and steel wire [7,20]. This crystallinity also allows cellulose to act as a nucleating agent in the PLA matrix, increasing PLA/CNC crystallinity and affecting mechanical properties [7].

Kumar et al. evaluated the properties of 1, 2, 5, and 10 wt % PLA/CNC composites [7]. They indicate that the crystallinity, melting temperature, and glass transition temperatures change with the introduction of CNCs. The optimal incorporation, 1 wt % CNCs in PLA, had the highest cold crystallization and an increased elastic modulus of 4550 MPa compared to 3030 MPa for neat PLA FDM-printed materials [7]. Importantly, all samples, regardless of CNC incorporation, retain a constant and steady viscosity at the extruder shear rate, allowing for 3D printing without clogging the extrusion head. The yield strength of 1 wt % CNC also increased to 61.07 MPa compared to 51.4 MPa for neat PLA. However, there was a 2.8% drop in strain at break for 1 wt % PLA/CNC compared to neat PLA, indicating that the orientation of the CNCs allows for absorption of the load, but reduced strain [7].

Other forms of cellulose have also been incorporated into PLA to enhance mechanical properties [38]. For example, previous studies indicate that incorporating cellulose nanowhiskers (CNWs) silylated with (3-mercaptopropyl) trimethoxysilane into PLA nanocomposites increases the elongation at break 250.8% compared to neat PLA [39]. The silylation reaction functionalizes the hydroxyl groups on cellulose, increasing CNW compatibility with PLA. Functionalization also modifies the usable temperatures of the PLA/CNW nanocomposite. The addition of silane A-151 increased the T_max_ to 304.4 °C [24]. Importantly, the concentration of A-151 must be high enough, at least 8 wt %, to fully coat the CNWs and create an even surface. While silylation increased the compatibility of the PLA and CNWs, the tensile strength and tensile modulus decreased minimally with increasing silane concentration. However, the elongation at break increased significantly (from 12.3% to 213.8%) with increasing silane concentration. The stiffness also increased with silane addition. In general, the tensile strength and tensile modulus increase with silylation of CNWs; however, the thermal properties, including the glass transition temperature, crystallinity, melting temperature, and crystallization temperature, decrease after silylation.

Cellulose nanofibers (CNFs) have also been investigated in the context of PLA 3D printing. Interestingly, the method of 3D printing affects the mechanical properties of CNF-containing PLA nanocomposites [40]. Specifically, the strength and modulus of FDM-printed neat PLA is 49 and 41% lower than its compression molded counterparts. With the addition of CNFs, at just 1 wt %, the strength and modulus of 3D-printed PLA/CNFs increased by 84% and 63% compared to PLA, respectively [40]. Incorporating CNFs into PLA significantly decreased voids and facilitated nucleation and crystallization, leading to increased matrix crystallinity. This higher crystallinity and reduction of defects caused overall increased mechanical properties.

Another cellulose-based derivative, industrial hemp hurd (HH), is a lignocellulose byproduct of the cannabis industry. HH contains 18–24% hemicellulose, 21–24% lignin, and 48% cellulose [41]. Xiao et al. introduced HH into PLA in the presence of toughening agent poly(butylene adipate-co-terephthalate) (PBAT) and interfacial modifier ethylene-methyl acrylate-glycidyl methacrylate (EGMA) terpolymer. The polymer matrices were all 87 wt % PLA and 13 wt % PBAT with 10–40 phr HH. Increasing HH concentration increased the complex viscosity, indicating a decrease in melt flow and 3D printability. The PLA/HH nanocomposites also showed increased shear-thinning behavior with increasing HH loading, which assists in 3D printability. Storage and loss moduli also increased with increasing HH content, especially at low frequencies, due to inhibition of chain mobility [41]. Contrastingly, the tanδ and angular frequency decreased with increasing HH loading. Interestingly, the addition of HH did not affect the thermal transitions such as T_g_ and T_m_ but did increase the cold crystallization temperature, indicating that increasing HH decreased crystallization enthalpy. With increased loading of HH the fracture surfaces showed less interfacial adhesion, indicating that HH decreased interfacial adhesion of polymer surfaces. HH also increased surface roughness and darkened the color of the composite. Overall, both the flexural strength and tensile strength decreased with increasing HH loading. Nevertheless, PLA/HH-30 had an impact strength comparable to commercial PLA FDM samples [41].

Poly(3-hydroxybutyrate) (PHB) has been incorporated into PLA/CNC nanocomposites through single-step blending with a crosslinking agent, dicumyl peroxide, which caused increased adhesion between the PLA and PHB phases and increased dispersion of CNCs. After 3D printing, the polymers align parallel to the extrusion direction indicating that FDM 3D printing changed the distribution and orientation of the CNCs. While the initial FDM printing of the nanocomposite caused some aggregation of CNCs, reprocessing those printed materials produced good dispersion of CNCs in the copolymer matrix [21].

PLA/PHB nanocomposites also facilitate introducing recycled cellulose in the form of pinewood fibers [42]. These PLA/PHB/wood composites are processable from 210 to 230 °C. This narrow window enables PLA/PHB melting without degradation of the wood nanofibers. This low print temperature not only makes the polymer desirable for home printing applications but also lowers the energy, and therefore cost, associated with printing. While CNCs improve mechanical properties, the PLA/PHB/wood nanocomposites show a 35% decrease in strength and 41% decrease in stiffness compared to neat PLA. The tensile strength was also low, not exceeding 21 MPa [42].

Adding plasticizers such as poly(ethylene glycol) (PEG) into PLA may increase ductility and toughness, but often decreases strength and stiffness [20]. These drawbacks may be mitigated by introducing nucleating agents, such as CNCs or CNFs, which increase the crystallinity of the PLA/PEG matrix and thus increase the strength and stiffness. Adding CNFs and CNCs gives significantly higher crystallinity than neat PLA or commercially available PLA/talc nanocomposites [20].

Solvent effects on CNC dispersion have recently been investigated in both amorphous and semi-crystalline PLA samples. Thermodynamic analysis identified dimethyl sulfoxide/tetrahydrofuran (DMSO/THF) as an optimal solvent system to incorporate CNCs into PLA because it can both dissolve the PLA and distribute the CNCs [43]. In semi-crystalline PLA nanocomposites, the CNCs distribute effectively but in amorphous nanocomposites the CNCs aggregate [43]. With the addition of CNCs, the storage modulus and complex viscosity increase. Significantly, residual solvent in the nanocomposite matrices caused dramatic decreases in complex viscosity (1 to 2 orders of magnitude) [43].

**Figure 4:**
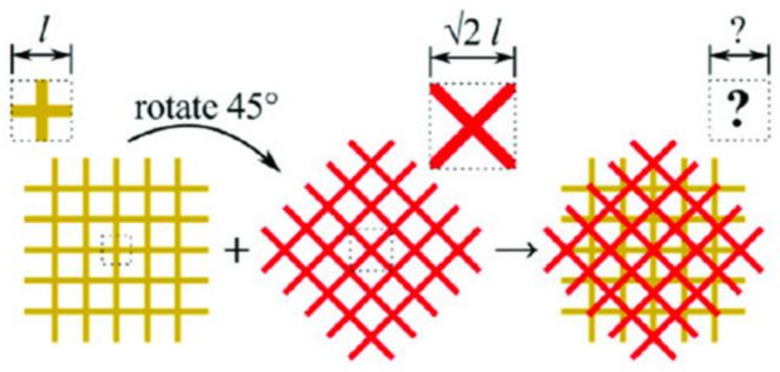
Depiction of rotation and crosshatched 3D printing pattern utilized to increase mechanical properties of 3D-printed materials. Reproduced with permission from [44], Wiley, 2020.

Cellulose in the form of wood fiber also increases the mechanical properties of PLA. Adding wood fiber to the PLA filament and subsequent FDM 3D printing produced materials with increased stiffness (18%), toughness (44%), thermal conductivity (23%), fracture strain (15%), strength (9%), and decreased density (10%) [44]. In FDM 3D printing, the printer typically prints line by line, with subsequent layers depositing directly on top of the previous layer. However, with the introduction of “isomixed” printing, a printing method where the printer prints crosshatched layers with each subsequent layer rotated by 45 degrees such that each new layer lies not perfectly on top but askew from each other as seen in Figure 4, there is an increase in properties. Isomixed microarchitectures of PLA/wood fiber nanocomposites show a 91% increase in stiffness and 48% increase in strength compared to neat PLA [44].

Cellulose, in the form of lignocellulosic fillers (LCFs), has also been incorporated into PLA/graphene nanoplatelet (GNP) nanocomposites to reduce the required GNP content and increase the mechanical properties of the materials. The introduction of both LCFs and GNPs allowed for good dispersion in the PLA matrix. Further, the introduction of these nanoparticles in combination increased nanocomposite stiffness up to 55%, elongation at break up to 35% and tensile strength up to 48% [45]. These results indicate that the introduction of GNPs and LCFs mitigate the reduced mechanical properties often found with natural fibers while strengthening the matrix. Varying the ratio of GNP to LCF modulated the degradation from 8% to 70% after just 900 h submersion in an alkaline environment and 1% to 30% in a neutral environment, indicating that this formulation can be tuned for many applications and degradation rates [45].

## 4. Metal-Based Additives

Metal additives can increase the mechanical and antibacterial properties of PLA nanocomposites. For example, Bayraktar et al. loaded silver nanowires into PLA, through solution mixing, as an antibacterial agent [46]. During processing, the suspended silver nanowires aligned in the direction of shear force homogeneously but broke down. Specifically, the shear force and heat required for 3D printing broke the nanowires into smaller particles but conserved the surface morphology. TGA showed that the nanowires influence the degradation of the PLA matrix: They increased the degradation temperature, increased crystallinity before printing, decreased T_g_, decreased crystallinity after printing, and did not change T_m_ [46]. Interestingly, the concentration of nanowires decreased after printing, indicating that some silver nanowires stuck to the inside of the polymer extrusion nozzle. Overall, the addition of silver nanowires added a barrier to degradation of the PLA/Ag nanocomposite while adding an antibacterial property, killing 100% of both *S. aureus* and *E. coli* for all concentrations of silver nanowires studied [46]. More recent studies on introducing silver nanoparticles indicate that composites with no significant change in bulk properties can be formed with an addition of 0.01–5 wt % silver nanoparticles. At all loadings of silver nanoparticles studied, these new PLA/Ag nanocomposites showed antimicrobial properties against *E. coli*, *P. aeruginosa*, and *S. aureus* [47].

Many industrial applications cannot use FDM-printed parts because fractures occur between layers due to poor interfacial adhesion and low surface quality [48]. Therefore, modifying FDM PLA nanocomposites to increase this surface quality may increase industrial use of these nanocomposites. Incorporating aluminum into nanocomposites produces air-cooled heat exchangers with high thermal conductivity at a low cost. These PLA/Al composites, when laser polished, show increased surface quality, decreased surface roughness, increased storage modulus, decreased loss tangent, increased tensile strength, and increased Young’s modulus [48]. These enhanced properties arise from the polymers becoming molten on the surface and reforming into a much more dense and interconnected structure during laser polishing.

**Figure 5:**
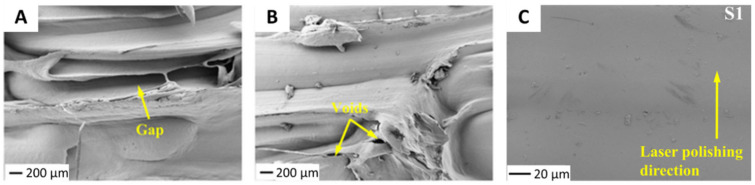
Poly(lactic acid) (PLA)/copper fiber surfaces (**A**,**B**) before laser treatment showing gaps, voids, and uneven surface morphology and (**C**) after 5 W laser treatment with a 175 µm beam. Reproduced with permission from [49], Wiley, 2020.

Similarly, copper fiber can be incorporated into a PLA matrix. Copper fiber/PLA composites contain uneven surface morphologies after FDM 3D printing due to the layer-by-layer addition, which often leaves ridges in the surface of printed materials. To ameliorate the effects of this surface structure, laser polishing is employed to melt the polymer matrix at the surface, producing a smoother surface [49]. The surface roughness decreases over 90% after laser treatment to 0.87 µm Sα with a 5 W laser of 200 µm (ideal parameters) as seen in Figure 5. This polishing also significantly improved the glass transition, storage modulus, Young’s modulus (34.2%), loss modulus, and tensile strength (52.98%) of Cu/PLA due to strong interfacial adhesion between PLA and Cu fibers after treatment [49].

Hybrid FDM (HFDM) methods incorporate metal meshes into the matrix of PLA. In these methods, a tensile bar is printed, wire mesh is laid on top of that piece, and a top layer is then printed, encasing the copper mesh into the 3D-printed materials and forming a composite. These composites show increased resistance to tearing, fracture load, and flexural strength [50]. Interestingly, in three-point flexural tests neat PLA shows a clean break at the point where force is applied. In contrast, PLA/Cu HFDM materials experience striations (small cracks) throughout the matrix rather than a clean break at the point of force application [50].

Introducing wood, as particleboard wood flours (PWF), into PLA nanocomposites would create a fully biodegradable and strong nanocomposite while maintaining the aesthetic appearance of wood. However, introducing PWF into PLA does not produce a glossy appearance or antibacterial properties. Therefore, micrometer copper–zinc (mCu–Zn) alloys have been introduced [51]. First, PWF was treated with hydrogen peroxide under alkaline conditions to remove cellulose, which may degrade at high processing temperatures and cause the material to discolor or lose strength [51]. Maximum mechanical properties occurred when 5 wt % PWF was introduced into the PLA matrix. The addition of mCu–Zn increased the thermal properties of PLA/PWF nanocomposites and, with an introduction of 2 wt % mCu–Zn, and produced glossy, antibacterial nanocomposites [51].

Adding magnesium into PLA matrices can improve mechanical properties while remaining completely biodegradable and bioresorbable [52]. Adding magnesium also increases the PLA degradation rate, reducing the time it exists in the environment [53,54,55,56]. Importantly, homogenous magnesium distribution is not readily achieved, but increases with addition of vitamin E. This PLA/Mg/vitamin E filament enabled printing of surgical implant screws [52].

More recently magnesium has been incorporated into PLLA matrices in the form of MgO nanowhiskers. The functionalization of magnesium to magnesium oxide increased the dispersion of the nanoparticles and improved the mechanical properties [57,58]. These MgO nanowhisker-containing materials undergo in vivo degradation, which is modulated by the amount of MgO incorporated and the molecular weight of PLLA in the matrix. With the incorporation of MgO, an increase in bone cells occurs, likely due to the positive effect of the Mg^2+^ degradation product on osteogenesis [57]. This finding indicates that increased MgO content increased desirable properties for bone implants.

Magnesium oxide nanowhiskers modified with stearic acid improve strength and toughness of PLLA nanocomposites. The addition of stearic acid increases PLA compatibility, and while some aggregation of nanowhiskers in the PLLA nanocomposite occurs, no significant cavities resulted [59]. At a nanowhisker content of 1 wt %, the PLLA/MgO nanocomposites increase the tensile strength by 17% and Young’s modulus by 78% compared to neat PLLA. The introduction of MgO nanocomposites also promoted nucleation, increasing crystallinity and decreasing the T_cc_ [59].

To further understand in vivo and in vitro degradation, the shape effect of magnesium oxide (nanowhiskers versus nanoparticles) was explored [60]. Introducing MgO in any form significantly accelerated degradation by accelerating water uptake into the polymer. After water uptake, the polymer first loses the nanocrystalline regions, followed by degradation of the crystalline regions [60]. Importantly, the addition of nanowhiskers increased the degradation rate more than the introduction of nanoparticles. In previous studies, modulating the MgO nanowhiskers content in PLLA nanocomposites modulated the degradation rate with an increase in degradation occurring with increased MgO nanowhisker content [61]. The introduction of MgO nanowhiskers also increased the cytocompatibility of the PLLA composites as seen through increased cell counts, consistent with previous studies on unfunctionalized magnesium [55,61].

**Figure 6:**
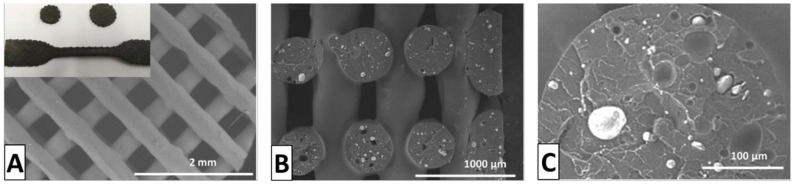
Compatibilized magnesium nanoparticle PLA filaments. (**A**) Fused deposition modelling (FDM) 3D-printed scaffold, (**B**) layer structure of scaffold, (**C**) cross section of individual layer showing magnesium dispersion. Reproduced with permission from [53], Elsevier, 2020.

To further compatibilize magnesium with the PLA matrix, the surface of Mg nanoparticles has been modified with adsorption dispersants. Polyethyleneimine (PEI) and cetyltrimethylammonium bromide (CTAB) were added in aqueous suspension and facilitated the ionic interactions of PLA and Mg nanoparticles. The introduction of PEI and CTAB enabled good dispersion in the PLA matrix as seen in Figure 6 [53]. In 3D-printed samples, PEI and PLA become covalently bonded through amide bonds, likely resulting from the temperatures required for printing. The addition of surface-functionalized Mg nanoparticles increased the Young’s modulus and strength when compared to neat PLA. The matrix then improves the extrusion and printability of PLA by compatibilizing the magnesium and PLA and eliminating nozzle clogging [53].

## 5. Continuous Fibers

**Figure 7:**
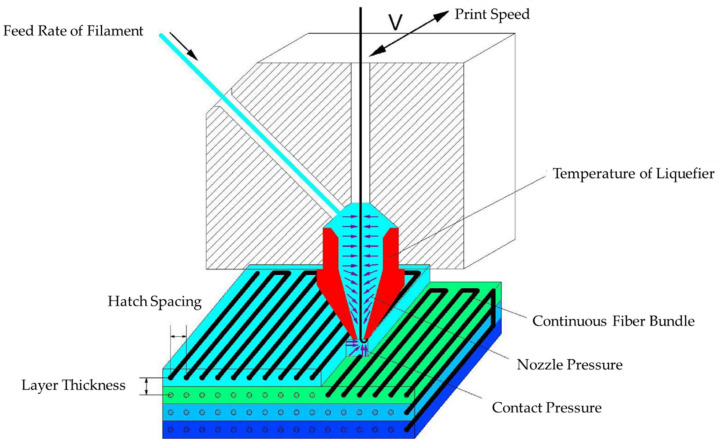
FDM 3D printing apparatus with continuous fibers. Reproduced with permission from [63], Elsevier, 2016.

Recently, introducing continuous fibers into PLA matrices has been explored. These fibers made of carbon, flax, pineapple leaf, and others are strong uninterrupted materials, which can increase mechanical properties [62]. Adding these continuous fibers, if used in conjunction with FDM 3D printing, occurs in the extrusion head when the polymer matrix is molten. Figure 7 depicts one common method for entrapping continuous fibers where the molten polymer completely surrounds the fiber [63].

Carbon fiber-reinforced (CFR) PLA matrices have the capacity for increased materials properties. Importantly, effectively impregnating the carbon fiber into the PLA matrix and forming the composite material requires a temperature of 200–230 °C. This temperature allows adequate flow of PLA while limiting the liquid-like and gravity-dependent flow properties when exiting the feed head [63]. The addition of 27% carbon fiber in CFR PLA composites increased the flexural strength of the composite to 335 MPa and flexural modulus to 30 GPa. Decreased layer thickness in FDM printed parts, and thus increased contact pressure and increased lamination between layers, caused increased flexural strength [63].

New methods for CFR PLA formation require optimization of printing parameters. The printing of continuous fibers requires that the carbon fiber remain at optimal tensions to reduce problems from wavy fibers in FDM 3D-printed materials [64]. For this reason, an “embedding on the component” method has been employed. This method allows the computer to control the PLA filament and carbon fiber filament speed when entering the extrusion head. This method, along with PVA pretreatment of carbon fibers, has increased the tensile strength by 35% and bending strength by 108% compared to neat PLA [64]. The failure methods of this composite, when exposed to external stress, are primarily delamination-induced matrix cracking and delamination. Importantly, the PLA/carbon fiber interface is well bonded with little delamination before cracking of the PLA matrix [64]. Interestingly, continuous carbon fiber-containing PLA composites printed with square-shaped nozzle outlets have increased fracture toughness and decreased bonded areas compared to those printed with a circular extrusion nozzle [65].

**Figure 8:**
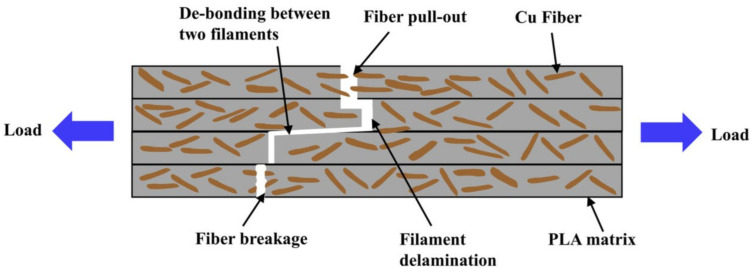
Depiction of fibers in FDM 3D-printed material and the different defects that occur with stress including fiber pull-out, filament delamination, debonding, and fiber breakage. Reproduced with permission from [49], Wiley, 2020.

Further insights into the strengthening mechanism of CFR PLA composites came through studying the fiber-matrix bonding interface strength, relative fiber content, and failure form of FDM-printed materials through field emission scanning electron microscopy [66]. With increased layer height (from 0.2 to 0.4 mm) and extrusion width (0.86 to 1.5 mm), tensile properties decreased, largely due to the increased concentration of PLA in comparison to carbon fiber content. Tensile properties also decreased slightly with increased temperature (from 190 to 230 °C) and increased feed rate (50 to 400 mm/min) [66]. Interestingly, the main failure mode for continuous carbon fiber-reinforced PLA is fiber pull-out caused by interface failure, meaning that the carbon fiber releases from the PLA matrix and pulls through the polymer rather than fracturing with the PLA as depicted in Figure 8. Pull-out is directly affected by the cohesion of the carbon fiber/PLA interface, indicating that increased adhesion would increase tensile properties [66].

Other continuous fibers also lend themselves to incorporation into polymer matrices. Flax fibers are among the strongest natural fibers and therefore a good option for introducing into PLA while maintaining biodegradability [67]. When coated with PLA, the twisted nature of the flax yarn causes imperfect impregnation and fiber-rich areas with minimal bonding to the PLA matrix. Even though imperfect binding occurred between the yarn and PLA matrix, the resulting composite exhibited good tensile behavior [67]. Further probing of slicing parameters, including layer height and number of layers, on FDM-printed parts determined that these parameters affect the properties of the continuous flax fiber/PLA composites [68]. Decreasing layer height from 0.6 to 0.2 mm increased stiffness and strength by over 210%, decreased porosity, and decreased layer thickness. Interestingly, layer thickness is determined, in part, by the width and compressibility of the flax yarn as the flax yarn must be within the PLA matrix. Increasing the number of layers (1 to 10) increased compaction ratio, tensile modulus (over 50%), and tensile strength (over 73%) [68]. For manufacturing, curved and bending 3D-printed parts, the introduction of continuous flax fibers increased the curved bending force by 39% and stiffness by 115% for FDM materials compared to the flat-slicing method of 3D printing (printing with supports where each layer is printed flat rather than printing onto an already curved area) [69].

Other natural fibers have also been incorporated into PLA matrices. Pineapple leaf fiber is currently used for wallpaper, textiles, and rope. This fiber has a diameter ranging from 0.1 to 0.5 mm, length ranging from 55 to 75 cm, tensile strength between 170 and 1627 MPa, and an elongation before break of 0.8 to 2.4% [70]. Interestingly, the pineapple leaf fibers do not require functionalization and can feed through a typical FDM setup with the PLA filament rather than the embedding method required for other continuous fibers depicted in Figure 8. Increasing the feed rate increased the tensile strength and decreased the printing time at 200 and 205 °C. At 210 °C increasing the feed rate decreased the tensile strength. Increasing the extrusion temperature from 200 to 210 °C increased the tensile strength from 85.30 MPa to 101.51 MPa at a feed rate of 15 mm/s [70].

The addition of continuous fibers brings into question the recyclability and biodegradability of the resulting new composites. Recycling the continuous carbon-impregnated PLA through melting and then introducing virgin PLA increased the mechanical properties and increased flexural strength by 25% compared to the original 3D-printed material, due to the improved interfacial properties compared to the first printing process. Since the continuous fiber does not break though tensile testing and does not pull out, this recycling process is a non-downgrade recycling process, the first for continuous fibers [71]. With good material recovery, 100% carbon fiber and 73% PLA, this recycling process represents an exciting step forward toward recyclable, high-quality PLA composites. While this process is by no means low energy (67.7 MJ/kg for recycling and 66 MJ/kg for remanufacturing), it is a promising approach towards recyclable materials [71].

Fiberglass, another widely used industrial continuous fiber, is used in everything from boat hulls to wind turbine blades. Recently, these turbine blades have been broken down, after decommissioning of the turbine, to collect the fiberglass fibers inside. These fibers are then embedded in PLA matrices for FDM 3D printing [72]. Addition of 5 wt % recycled fiberglass in FDM-printed materials increased the elastic modulus 16% and strength 10% compared to neat PLA. The Young’s modulus also increased 8%, to 3.35 GPa [72]. This increase in mechanical properties indicates that the PLA/fiberglass composites are a viable option for repurposing fiberglass materials.

## 6. Carbon-Based Additives

Carbon-based additives can be incorporated into PLA to increase thermal/electrical conductivity and mechanical properties [73]. The most common carbon-based additives include carbon nanotubes (CNTs), multiwalled carbon nanotubes (MWCNTs), and graphene nanoplatelets (GNPs) [8,74]. Such modifications, however, increase the risk that the additive will reduce or eliminate the biodegradability of the PLA matrix. Another concern, especially with carbon-based additives, is that the degradation byproducts contain hazardous particles. However, a study by Ivanov et al. indicates that PLA/carbon nanocomposites biodegrade into safe byproducts under standard degradation conditions [11,75,76,77].

Adding CNTs—cylindrical sheets of graphene—into PLA affects the mechanical and conductive properties of the material. Including CNTs in any concentration reduces the melting temperature of PLA and inhibits crystallization, indicating that adding CNTs adversely affects FDM printing. While CNTs may hinder the printing process, the addition of only 6 wt % CNT increased the tensile strength of PLA/CNT 64% and flexural strength 29% [78]. The electrical resistivity of these nanocomposites also increased to between 10^12^ Ω/sq and 10^2^ Ω/sq with CNT content from 0 to 8 wt % [78]. To achieve the best electrical conductivity in the PLA/CNT nanocomposites studied, a high extrusion nozzle temperature, large layer thickness, and small filling velocity were employed [78]. In a related study, increasing the CNT content was found to increase the degree of crystallinity of PLA/CNT nanocomposites, likely due to the small defects on CNT surfaces, which become nucleating sites. The addition of 3 wt % CNTs produced a material with a surface resistivity of 10^5^ Ω/m^2^, indicating that these printed materials have resistance similar to the human body [79]. This resistance proves that the CNTs are well ordered after printing and that these PLA/CNT nanocomposites may be usable as thermal resistance plastics [79].

MWCNTs—CNTs with multiple concentric layers—and GNPs (graphene sheets) have been introduced into PLA through solution blending and melt mixing. In both melt-mixed and solution-blended samples, increasing the GNP content caused aggregation. In mixed GNP/MWCNT materials, this aggregation likely results from the MWCNTs adsorbing onto the surface of GNPs. Agglomerates of approximately 50 nm appear even at the lowest studied concentration of GNPs/MWCNTs in solution-blended nanocomposites. However, the melt-mixed nanocomposites of PLA/1.5 wt % GNP/4.5 wt % MWCNT show no aggregation [74]. Formation of the PLA/GNP/MWCNT nanocomposites into filaments is a necessity for FDM printing and thus the nanocomposite must retain good rheological and mechanical properties during extrusion into a filament. The viscosity of nanocomposites doubled in the 6 wt % materials compared to neat PLA. To achieve electrical conductivity, PLA nanocomposites require 1.5–3 wt % and 3–6 wt % for MWCNTs and GNPs, respectively. Interestingly, the electrical conductivity was greater in melt-mixed nanocomposites compared to their solution-blended counterparts because of the increased surface contact area of nanoparticles when introduced via melt-mixing. Overall, the melt-mixed polymers gave better rheological properties, higher electrical conductivity, stronger electromagnetic shielding, and were easier to process than those produced by solution blending [74]. Further examination of the thermal and electrical conductivity of GNP and MWCNT nanocomposites determined that the addition of 12% GNPs increased thermal conductivity by 263%, compared to 99% with MWCNTs and 190% with a bifiller system [8]. Improved thermal properties were also seen with MWCNT, GNP, and a bifiller composition of up to 4.54 S/m, 6.57 S/m, and 0.95 S/m respectively [8].

Batakliev et al. found that better mechanical properties occur with the addition of 6 wt % MWCNTs or GNPs compared to 1.5, 3, 8, and 12 wt % [80]. Initially, the Young’s modulus of GNP/PLA nanocomposites remains largely unchanged for all GNP-loaded samples due to their high degree of exfoliation. Compared to neat PLA, the tested nanocomposites showed a 20–50% increase in elasticity and 30–50% increase in hardness resulting from the good dispersion of GNPs and MWCNTs [80]. When compared to neat PLA, the best mechanical properties in this study come from bifiller systems with equal concentrations, specifically a 12 wt % total concentration (6 wt % GNP/ 6 wt % MWCNT) [80].

Introducing carbon-based additives also enables printing electrically conductive parts. To conduct electricity the nanocomposite must pass the percolation threshold, the transition from a conductive to insulating polymer, while remaining viable for 3D printing [11]. MWCNT-containing systems did not show aggregation at 6 wt %; however, GNP systems at the same concentration showed agglomeration, likely due to π–π and Van der Waals interactions between the graphene sheets. Bifiller PLA/GNP/MWCNT materials have increased dispersion, which increased the electrical conductivity 7–8 decades compared to neat PLA to a value of 8.4 × 10^−3^ S/m. Importantly, all samples over 3 wt % total monofiller or bifiller concentration pass the percolation threshold, allowing for electrical conductivity [11]. If heat is not dissipated in electrically conductive materials, the lifetime of the printed materials diminishes. Thermal conductivity depends on several factors including heating temperature, polymer crystallinity, and orientation of macromolecules [11]. In these polymer nanocomposites, the thermal conductivity increased linearly with increasing filler content, while thermal diffusivity also increased [11].

While previous nanocomposites containing MWCNTs and GNPs showed enhanced mechanical properties, the agglomeration of nanoparticles causes issues such as nozzle clogging or inconsistent mechanical properties throughout the matrix [81]. Oxidizing the MWCNT surface introduces hydroxyl groups, leading to better incorporation into the PLA matrix. The low flow index and high initial viscosity, even at a low loading of 1.5 wt %, indicate that the oxidized MWCNT-containing nanocomposites show high dispersion. Interestingly, MWCNTs with higher specific surface area, 250–300 m^2^/g compared to 110 m^2^/g, have about two times better dispersion. In contrast, GNP nanoparticles show low dispersion and cause 12% (aspect ratio 500) and 25% (aspect ratio 240) reductions of strength and elongation [81]. In contrast, the strength and elongation for oxidized MWCNT samples only slightly decreased (at 12 wt % loading) [81]. The thermal conductivity of PLA/GNP nanocomposites increased with the addition of GNPs more than with the introduction of oxidized MWCNTs [81]. This data indicates that oxidation of MWCNTs increases dispersion while decreasing the mechanical properties of the nanocomposite.

**Figure 9:**
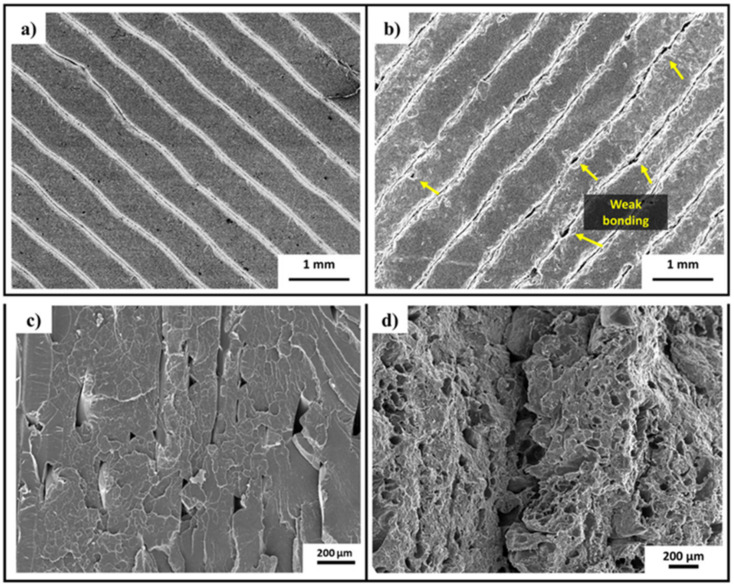
Integration of graphene nanoparticles into FDM 3D-printed PLA. (**a**) Neat PLA after FDM 3D printing. (**b**) PLA/graphene nanocomposite after FDM 3D printing. (**c**) PLA and (**d**) PLA/graphene fractured cross-sectional microstructure. Reproduced with permission from [82], Wiley, 2018.

The creep behavior—the tendency to deform under stress—of 3D-printed PLA nanocomposites impacts their viability and long-term strength. Bustillos et al. studied PLA/graphene 3D-printed materials that contained a good dispersion of GNPs, porous structure, and a well-defined pore-size gradient as depicted in Figure 9 [82]. The PLA/GNP printed materials showed a higher cooling and nucleation rate compared to neat PLA due to the introduction of GNP nanoparticles with higher thermal conductivity. The fast cooling causes thermal strain mismatch between the GNPs and PLA matrix, causing poor interlayer and intralayer bonding. The printed material and filament also show a lower T_g_ compared to neat PLA; however, the 3D-printed material had a 12% higher T_g_ than its corresponding filament, indicating the 3D printing process increases the interactions between graphene and PLA. GNP-containing samples also showed a higher nucleation rate and a corresponding decrease in the T_cc_. Crystallinity also decreased about 8% in the 3D-printed PLA/GNP material, which was attributed to the rapid cooling and therefore increased amorphization [82]. The GNP-reinforced samples also showed an increase in hardness (18%), elastic modulus (11%), and resistance to displacement (25%). The permanent deformation also decreased: After elongation, neat PLA samples only recover 25% compared to the 43% recovery of the PLA/GNP nanocomposite. Overall, adding GNPs to the PLA matrix caused a 20.5% decrease in creep displacement, 14% increase in wear resistance, and overall increase of mechanical properties [82].

Incorporating additional chemicals, such as L-arginine, into PLA/GNP matrices decreases phase separation and increases GNP dispersion in the matrix. For example, at 2 wt % L-Arg/GNP loadings, limited agglomeration occurs; however, increasing GNP concentration increases agglomeration. 3D-printed materials produced from solution-mixed filaments of these composites [83]. The fracture surfaces of the 3D-printed structures occur in the interlayer space, indicating good adhesion and interlayer homogeneity. The elongation at break of these PLA nanocomposites increased from 8.3% to 14.8%, indicating that the introduction of GNPs decreased the brittleness of the polymer and transformed it into a more ductile material by transferring stress and preventing cracking. Both the flexural strength and tensile strength also increase with increased L-Arg [83]. The enhanced mechanical properties are presumably caused by the polar groups on the GNP surface; however, at GNP concentrations greater than 2 wt %, increased concentration decreases mechanical properties. When GNP content is held constant, the matrices containing L-Arg have higher T_g_, stronger interfacial binding, and lower T_m_. The crystallinity of 2 wt % PLA/L-Arg/GNP samples also increased to 16.74%, almost six times that of neat PLA. At the optimal loading of 2 wt %, the degradation temperature increased by 60 °C [83].

The printing parameters, including infill and layer thickness, also affect mechanical properties of PLA/GNP FDM-printed materials. Tensile strength and flexural strength increased with increasing layer thickness and infill to a maximum of 33.7 and 60.9 MPa, respectively, with a layer thickness of 0.27 mm and infill of 78% [84]. The impact energy reached a maximum value of 70.9 J/m with an infill of 50% and layer thickness of 0.20 mm [84]. Interestingly, the impact energy decreased with increasing infill and layer thickness until the midpoint of each factor after which it increased.

Modification of MWCNTs and graphene through oxygen plasma etching (OPE) increases oxidation and exfoliation on their surface, which leads to better incorporation and increased in vivo viability. Introducing these OPE MWCNTs into PLA decreased mechanical properties, increased cell adhesion, and increased cell viability [85]. While the introduction of graphene oxide and MWCNT oxides decreased the elongation-at-break and tensile strength compared to neat PLA, the mechanical performance remained sufficient for bone implants. Importantly, the introduction of graphene oxide and MWCNT oxide nanoparticles significantly increased the osteoblast ALP cell activity in vitro and significantly increased bone cell activity in vivo [85].

While the addition of carbon nanoparticles into PLA matrices has increased the mechanical properties significantly in many cases, a key barrier remains to FDM-printed part acceptance in industrial applications: fractures at the layer interface. Interlayer cracking accounts for a large portion of breaks in FDM parts and therefore is one of the key reasons that FDM parts are not used industrially. Microwave irradiation can strengthen the interlayer adhesion after printing by melting the layers together, increasing the fracture strength by 275% [86].

## 7. Other Additives

Additives for PLA reinforcement are not limited to the materials previously described. Other additives including nanoclay, silica, other polymers, and various biodegradable bioderived powders have been incorporated into PLA to form biodegradable and well-dispersed PLA nanocomposites.

Nanoclay has been added to PLA to enhance its properties. These samples, PLA 4032D (T_m_ 170 °C, 1.5% D-isomer) and PLA 2003D (T_m_ 150 °C, 4% D-isomer), were processed at varying nozzle temperatures to determine the effect of nozzle temperature on clay-containing nanocomposites at different crystallinities [9]. Adding nanoclay increased the storage modulus of all samples, indicating the effectiveness of the nanoclay in strengthening the composite. Specifically, dynamic mechanical analysis (DMA) showed an increase in the storage modulus at 35 °C of 8 and 23% for PLA 4032 and PLA 2003 nanoclay composites. Nanoclay also acted as a nucleating agent, as shown by differential scanning calorimetry (DSC), and increased the thermal stability of composites. All printed samples were largely amorphous. Printing temperatures, when increased, increased the transparency of the PLA/clay nanocomposites. The increased nozzle temperature also increased brittleness of 3D-printed parts.

Adding silica, collected as sand from local beaches, leads to enhanced thermostatics, which increases handling and quality performance of thermoplastic polymers. Silica, one of the most abundant inorganic ceramics, is a good choice for incorporation into polymer matrices due to its low cost and good mechanical properties [87]. Incorporating silica during recycling not only increases the tensile strength to 121.03 MPa (with a 10 wt % addition) but also increases toughness, yield stress, and ductility [87]. The improved mechanical properties promote recycling of PLA while retaining their biodegradability.

Lyu et al. took an alternative approach to toughen the PLA matrix [88]: incorporating poly(butylene-adipate-co-terephthalate) (PBAT) into PLA-g-GMA (PLA grafted with glycidyl methacrylate) and PLA systems. PLA-g-GMA was added as a compatibilizer to stabilize the interface between the PLA and PBAT. All samples containing PLA-g-GMA compatibilizer showed an island phase morphology indicating increased homogeneity and surface compatibility. Samples without PLA-g-GMA compatibilizer had low viscosity with easily seen crystalline sections, indicating that without compatibilizer PBAT and PLA are completely incompatible and, therefore, PLA cannot hinder the PBAT crystallization. With the addition of only 10 wt % compatibilizer the viscosity increased and no PBAT crystalline region were visible.

The introduction of secondary polymers, such as polyhydroxybutyrate (PHB), enables mechanical properties to be tuned without reducing biocompatibility or biodegradability. PHB, a biocompatible, biodegradable, and sustainable polyester, is compatible with PLA [89]. Importantly, the amount of water absorbed into the polymer matrix from the environment affects 3D printability by affecting the viscosity. Also, if parts are dried after printing at 80 °C for 60 min, the materials become much more stable than their non-dried counterparts. Interestingly, printing shape also affects long term stability and material properties [89].

Polyhedral oligomeric silsesquioxane (POSS), another biocompatible and non-toxic polymer nanoparticle, also increases the flexural strength (22%), flexural modulus (9%), and fracture toughness (117%) of 3D-printed PLA/POSS nanocomposites compared to neat PLA [90]. PLA/POSS nanocomposites were formed through melt-mixing of pre-dried samples and then extrusion and FDM 3D printing, resulting in a good distribution of nanoparticles in the PLA matrix. Importantly, the addition of POSS allowed the samples to remain in a phosphate buffered saline solution at 37 °C for 120 days with little mechanical or physical deterioration, suggesting their potential use for longer-term medical implants [90].

Eggshell powder, functionalized with calcium phenylphosphonate, provides nucleation sites when incorporated into PLA matrices. This addition of eggshells, in combination with PEG plasticizer, occurs through melt blending [91]. Incorporating both functionalized eggshell and PEG into the PLA matrix produces a significantly increased nucleation and crystallization rate [91]. Further investigation into the addition of eggshell into PLA showed that eggshells modified with CaO-oleic acid show good dispersion and higher impact strength and elongation at break compared with unmodified or propionic acid modified eggshell composites [92]. These materials also show wider melt-processing windows than neat PLA [92]. Further analysis indicated that the incorporation of eggshell into PLA increases the Young’s modulus, thermal resistance, and plasticity [93].

## 8. Conclusions

PLA is an important biodegradable polymer produced from some of the most renewable feedstocks available. While PLA is useful in FDM 3D printing, its drawbacks—brittleness, poor thermal stability, low crystallization, low elongation at break, poor impact strength, low heat distortion temperature, and limited drawability—reduce its prevalence as an industrial material. Therefore, additives have been incorporated into PLA to form nanocomposites with enhanced mechanical, electrical, or thermal properties.

Cellulose is commonly incorporated into PLA matrices to enhance the mechanical properties while maintaining complete biodegradability. Key findings include:

- Silylation of cellulose increases the cellulose–PLA compatibility and results in increased tensile strength and tensile modulus but decreased glass transition temperature, crystallinity, melting temperature, and crystallization temperature [24].
- Adding cellulose decreases the processable temperatures due to cellulose thermal degradation [24].
- The incorporation of CNFs into PLA decreases voids, increases crystallinity, and decreases defects during the FDM 3D printing process [40].
- When aggregation occurs with cellulose-based additives, recycling and reprinting that material may lead to good dispersion [21].
- Printing in an “isomixed” orientation produces materials that are stronger and have increased stiffness [44].

Metal-based additives have also been explored because of their ability to increase strength, promote bone cell growth, and prevent bacterial growth. The incorporation of various metals, including silver, aluminum, copper, and magnesium, has increased mechanical and antibacterial properties. Specifically:

- At all concentrations, PLA/Ag nanowire nanocomposites show antimicrobial properties against *E. coli, P. aeruginosa*, and *S. aureus* [47].
- PLA/Al nanocomposites show increased surface quality, decreased surface roughness, increased storage modulus, decreased loss tangent, increased tensile strength, and increased Young’s modulus after laser polishing [48].
- The introduction of mCu-Zn into PLA/wood flour nanocomposites increases the aesthetic appearance by creating a glossy finish while also increasing the antibacterial properties [51].
- The distribution of magnesium nanoparticles increased with the addition of vitamin E, addition of stearic acid, the oxidation of the magnesium to magnesium oxide, or the functionalization of magnesium with PEI and CTAB [53].
- The Mg^2+^ ions released during PLA/MgO degradation increased bone cell growth [57].

Continuous fibers, another PLA additive, are long strands of microscale fibers that allow for increased mechanical strength. While these fibers have incredible strength, they suffer from a lack of compatibility with the PLA matrix; however, many advances in continuous fibers have occurred, specifically:

- Carbon fiber-reinforced PLA shows increased flexural strength, flexural modulus, delamination between layers, and contact pressure, but decreased layer thickness [63].
- The failure modes of carbon fiber/PLA nanocomposites are delamination, delamination-induced matrix cracking, or pull out [64].
- Carbon fiber-reinforced PLA has greater fracture toughness when extruded through square shaped extruder nozzles rather than circular [65].
- Tensile properties decrease with increased layer height and extrusion width [66].
- Carbon fiber-reinforced PLA matrices are not only partially biodegradable but are almost fully recyclable [71].

Carbon-based additives—including carbon nanotubes (CNTs), multiwalled carbon nanotubes (MWCNTs), and graphene nanoplatelets (GNPs)—increase the thermal, electrical, and mechanical properties of PLA nanocomposites. Key findings include:

- Increased CNT content increases degree of crystallinity in PLA/CNT nanocomposites [79].
- Nanocomposites of PLA/1.5% GNP/4.5% MWCNT show no aggregation and are, therefore, viable for FDM 3D printing [74].
- GNP addition increases thermal conductivity more than adding the same concentration of MWCNTs [80].
- Oxidizing MWCNT surfaces enables better incorporation into the PLA matrix with limited agglomeration [81].
- Introducing GNPs decreases creep, decreases wear resistance, and increases overall mechanical properties of PLA [82].
- L-arginine acts as a compatibilizer for GNPs and PLA, increasing the dispersion of GNPs in the PLA matrix [83].
- Oxygen plasma etching of MWCNTs increases the PLA/MWCNT nanocomposite cell adhesion in vivo, while maintaining adequate mechanical properties for bone implants [85].

While incorporating additives into PLA matrices has improved the mechanical properties of PLA nanocomposites, more advances are needed before PLA nanocomposites overtake petroleum-based materials in terms of ease of use and mechanical robustness. Current PLA composites often lack good dispersion and do not accommodate large loadings of nanomaterials in the PLA matrix. This problem has been partially addressed by functionalizing nanoparticles, which enables better surface contact area.

Moving forward, this field would benefit from further exploration of functionalized nanoparticles, specifically introducing functionalities that would stabilize the nanoparticle/PLA interface. Additionally, new additives and new combinations of additives should also be explored. Ultimately, these composites have the potential to replace environmentally harmful, non-degradable materials in a variety of applications, thereby offering a viable approach to a more environmentally responsible future.
